# 2-Butyl-1,3-diphenyl-2,3-dihydro-1*H*-naphtho[1,2-*e*][1,3]oxazine

**DOI:** 10.1107/S1600536808029383

**Published:** 2008-09-20

**Authors:** Yong Hua Li, Min Min Zhao, Yuan Zhang

**Affiliations:** a Ordered Matter Science Research Center, College of Chemistry and Chemical Engineering, Southeast University, Nanjing 211189, People’s Republic of China

## Abstract

In the title compound, C_28_H_27_NO, the oxazine ring adopts a half-chair conformation. The dihedral angles between the phenyl rings and the naphthyl ring system are 15.34 (1) and 76.51 (1)°.

## Related literature

For general background on oxazine compounds, see: Barker *et al.* (2006 ▶); Ren *et al.* (2001 ▶); Petterson *et al.* (1990 ▶); Peglion *et al.* (1997 ▶). For related structures, see: Alfonsov *et al.* (2007 ▶); Ji *et al.* (2005 ▶).
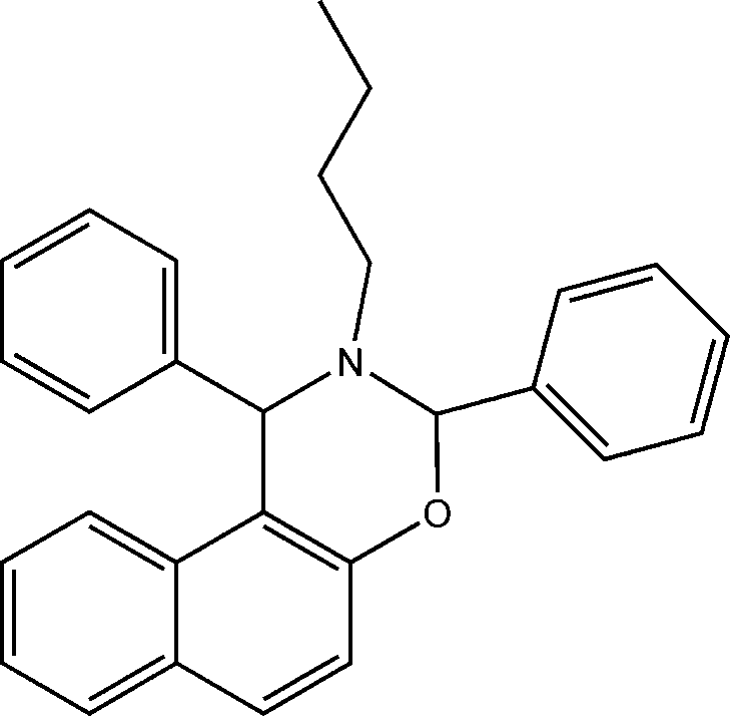


## Experimental

### 

#### Crystal data

C_28_H_27_NO
*M* *_r_* = 393.51
Triclinic, 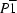

*a* = 8.8959 (15) Å
*b* = 10.7589 (16) Å
*c* = 11.8401 (18) Å
α = 96.219 (1)°
β = 98.366 (2)°
γ = 97.274 (2)°
*V* = 1102.8 (3) Å^3^
*Z* = 2
Mo *K*α radiation
μ = 0.07 mm^−1^
*T* = 293 (2) K
0.20 × 0.18 × 0.15 mm

#### Data collection

Rigaku SCXmini diffractometer
Absorption correction: multi-scan (*CrystalClear*; Rigaku, 2005 ▶) *T*_min_ = 0.965, *T*_max_ = 0.977
10108 measured reflections
4302 independent reflections
2352 reflections with *I* > 2σ(*I*)
*R*_int_ = 0.041

#### Refinement

*R*[*F* ^2^ > 2σ(*F* ^2^)] = 0.072
*wR*(*F* ^2^) = 0.262
*S* = 1.02
4302 reflections
272 parameters
H-atom parameters constrained
Δρ_max_ = 0.26 e Å^−3^
Δρ_min_ = −0.34 e Å^−3^

### 

Data collection: *CrystalClear* (Rigaku, 2005 ▶); cell refinement: *CrystalClear*; data reduction: *CrystalClear*; program(s) used to solve structure: *SHELXS97* (Sheldrick, 2008 ▶); program(s) used to refine structure: *SHELXL97* (Sheldrick, 2008 ▶); molecular graphics: *SHELXTL/PC* (Sheldrick, 2008 ▶); software used to prepare material for publication: *SHELXTL/PC*.

## Supplementary Material

Crystal structure: contains datablocks I, global. DOI: 10.1107/S1600536808029383/pv2101sup1.cif

Structure factors: contains datablocks I. DOI: 10.1107/S1600536808029383/pv2101Isup2.hkl

Additional supplementary materials:  crystallographic information; 3D view; checkCIF report

## Figures and Tables

**Fig. 1.**
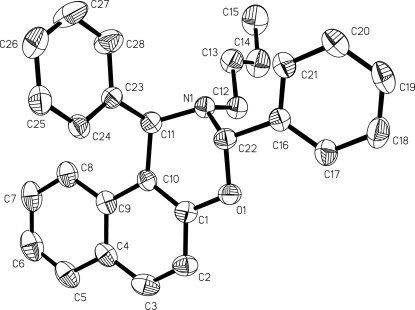
The molecular structure of the title compound, showing the atomic numbering scheme. Displacement ellipsoids are drawn at the 30% probability level.

## supplementary crystallographic information

### Comment

Continuing efforts have been made to synthesize oxazine compounds because they are widely used as antipsychotic agents (Barker *et al.*, 2006), antimalarial agents (Ren *et al.*, 2001) and serotonin, dopamine receptors agonists (Petterson *et al.*, 1990; Peglion *et al.*, 1997). We have prepared a novel compound, 2-butyl-1,3-diphenyl-2,3-dihydro-1*H*-naphtho[1,2-*e*][1,3]oxazine, (I), by the reaction of 2-naphthol, benzaldehyde and n-butylamine. In this paper, we present the synthesis and crystal structure of (I). The structures of some closely related compounds have been reported (Alfonsov *et al.*, 2007; Ji *et al.* 2005).

In the molecule of the title compound (Fig. 1), the oxazine ring adopts a half chair conformation. An intra-molecular interaction, C17—H15···O1, is observed in the crystal structure, but no inter-molecular hydrogen bonding was present. The dihedral angle between the C16–C21 phenyl ring and naphthyl system is 15.34 (1)° and the dihedral angle between the C23–C28 phenyl ring and naphthyl system is 76.51 (1)°.

### Experimental

The title compound was one of the products of the reaction between 2-naphthol, n-butylamine and an excess amount of benzaldehyde. Benzaldehyde (22.05 g, 0.208 mol) was added to a solution of 2-naphthol (15 g, 0.104 mol) in 20 ml 95% ethanol. n-Butylamine (7.65 g, 0.104 mol) was added dropwise with cooling to 273 K to this solution. The mixture was stirred at room temperature for 6 days and the precipitate was filtrated and washed with a small amount of 95% ethanol. The title compound was isolated using column chromatography (petroleum ether: ethyl acetate - 30:1). Single crystals suitable for X-ray diffraction analysis were obtained from slow evaporation of an ethanol solution.

### Refinement

H atoms were positioned geometrically, with C—H = 0.93, 0.98, 0.97 and 0.96 Å for aromatic, methine, methylene and methyl groups, respectively, and constrained to ride on their parent atoms with *U*_iso_(H) = *xU*_eq_(C), where *x* = 1.5 for methyl H and *x* = 1.2 for all other H atoms.

### Crystal data

**Table:**

C_28_H_27_NO	*Z* = 2
*M**_r_* = 393.51	*F*(000) = 420
Triclinic, *P*1	*D*_x_ = 1.185 Mg m^−^^3^
Hall symbol: -P 1	Mo *K*α radiation, λ = 0.71073 Å
*a* = 8.8959 (15) Å	Cell parameters from 2254 reflections
*b* = 10.7589 (16) Å	θ = 2.3–27.5°
*c* = 11.8401 (18) Å	µ = 0.07 mm^−^^1^
α = 96.219 (1)°	*T* = 293 K
β = 98.366 (2)°	Prism, colourless
γ = 97.274 (2)°	0.20 × 0.18 × 0.15 mm
*V* = 1102.8 (3) Å^3^	

### Data collection

**Table:**

Rigaku SCXmini diffractometer	4302 independent reflections
Radiation source: fine-focus sealed tube	2352 reflections with *I* > 2σ(*I*)
graphite	*R*_int_ = 0.041
Detector resolution: 13.6612 pixels mm^-1^	θ_max_ = 26.0°, θ_min_ = 2.3°
ω scan	*h* = −10→10
Absorption correction: multi-scan (*CrystalClear*; Rigaku, 2005)	*k* = −13→13
*T*_min_ = 0.965, *T*_max_ = 0.977	*l* = −14→14
10108 measured reflections	

### Refinement

**Table:**

Refinement on *F*^2^	Primary atom site location: structure-invariant direct methods
Least-squares matrix: full	Secondary atom site location: difference Fourier map
*R*[*F*^2^ > 2σ(*F*^2^)] = 0.072	Hydrogen site location: inferred from neighbouring sites
*wR*(*F*^2^) = 0.262	H-atom parameters constrained
*S* = 1.02	*w* = 1/[σ^2^(*F*_o_^2^) + (0.1501*P*)^2^] where *P* = (*F*_o_^2^ + 2*F*_c_^2^)/3
4302 reflections	(Δ/σ)_max_ < 0.001
272 parameters	Δρ_max_ = 0.26 e Å^−^^3^
0 restraints	Δρ_min_ = −0.34 e Å^−^^3^

### Special details

**Table:**

Geometry. All e.s.d.'s (except the e.s.d. in the dihedral angle between two l.s. planes) are estimated using the full covariance matrix. The cell e.s.d.'s are taken into account individually in the estimation of e.s.d.'s in distances, angles and torsion angles; correlations between e.s.d.'s in cell parameters are only used when they are defined by crystal symmetry. An approximate (isotropic) treatment of cell e.s.d.'s is used for estimating e.s.d.'s involving l.s. planes.
Refinement. Refinement of *F*^2^ against ALL reflections. The weighted *R*-factor *wR* and goodness of fit *S* are based on *F*^2^, conventional *R*-factors *R* are based on *F*, with *F* set to zero for negative *F*^2^. The threshold expression of *F*^2^ > σ(*F*^2^) is used only for calculating *R*-factors(gt) *etc*. and is not relevant to the choice of reflections for refinement. *R*-factors based on *F*^2^ are statistically about twice as large as those based on *F*, and *R*- factors based on ALL data will be even larger.

### Fractional atomic coordinates and isotropic or equivalent isotropic displacement parameters (Å^2^)

**Table:**

	*x*	*y*	*z*	*U*_iso_*/*U*_eq_	
C1	−0.0487 (3)	0.6705 (3)	0.1444 (2)	0.0520 (7)	
C10	−0.0728 (3)	0.7223 (3)	0.2512 (2)	0.0486 (7)	
C16	0.3447 (3)	0.8083 (3)	0.1275 (2)	0.0489 (7)	
C9	−0.2156 (3)	0.6833 (3)	0.2883 (3)	0.0551 (7)	
C11	0.0504 (3)	0.8228 (3)	0.3234 (2)	0.0522 (7)	
H5	−0.0016	0.8926	0.3505	0.063*	
C2	−0.1621 (3)	0.5819 (3)	0.0713 (3)	0.0604 (8)	
H6	−0.1439	0.5486	−0.0004	0.073*	
C22	0.2099 (3)	0.7704 (3)	0.1862 (2)	0.0505 (7)	
H7	0.2422	0.7124	0.2402	0.061*	
C4	−0.3288 (3)	0.5932 (3)	0.2146 (3)	0.0599 (8)	
C23	0.1350 (3)	0.7789 (3)	0.4296 (2)	0.0517 (7)	
C12	0.1042 (3)	0.9693 (3)	0.1822 (2)	0.0571 (7)	
H10A	−0.0048	0.9450	0.1546	0.069*	
H10B	0.1561	0.9711	0.1156	0.069*	
C21	0.4792 (3)	0.8709 (3)	0.1948 (3)	0.0621 (8)	
H11	0.4811	0.8926	0.2732	0.074*	
C3	−0.2976 (3)	0.5457 (3)	0.1064 (3)	0.0689 (9)	
H12	−0.3719	0.4880	0.0575	0.083*	
C8	−0.2484 (4)	0.7299 (3)	0.3967 (3)	0.0677 (9)	
H13	−0.1762	0.7890	0.4460	0.081*	
C24	0.1235 (3)	0.6555 (3)	0.4499 (2)	0.0560 (7)	
H14	0.0583	0.5941	0.3977	0.067*	
C17	0.3449 (4)	0.7784 (3)	0.0112 (3)	0.0641 (8)	
H15	0.2562	0.7370	−0.0362	0.077*	
C5	−0.4699 (3)	0.5539 (4)	0.2525 (4)	0.0777 (10)	
H16	−0.5451	0.4959	0.2046	0.093*	
C13	0.1311 (4)	1.1005 (3)	0.2498 (3)	0.0712 (9)	
H17A	0.0864	1.0966	0.3196	0.085*	
H17B	0.2408	1.1267	0.2724	0.085*	
C19	0.6094 (4)	0.8710 (3)	0.0332 (3)	0.0733 (10)	
H18	0.6979	0.8914	0.0018	0.088*	
C18	0.4781 (4)	0.8101 (4)	−0.0357 (3)	0.0799 (10)	
H19	0.4773	0.7898	−0.1141	0.096*	
C7	−0.3862 (4)	0.6891 (4)	0.4304 (3)	0.0834 (11)	
H20	−0.4062	0.7209	0.5021	0.100*	
C20	0.6101 (4)	0.9016 (3)	0.1482 (3)	0.0717 (9)	
H21	0.6991	0.9434	0.1952	0.086*	
C14	0.0657 (5)	1.1976 (4)	0.1856 (3)	0.0914 (12)	
H4	−0.0446	1.1733	0.1661	0.110*	
H22B	0.1067	1.1986	0.1141	0.110*	
C25	0.2064 (4)	0.6200 (4)	0.5459 (3)	0.0716 (9)	
H23	0.1957	0.5354	0.5574	0.086*	
C6	−0.4955 (4)	0.6004 (4)	0.3579 (4)	0.0896 (12)	
H24	−0.5874	0.5726	0.3821	0.108*	
C28	0.2381 (4)	0.8682 (3)	0.5083 (3)	0.0834 (11)	
H25	0.2524	0.9524	0.4960	0.100*	
C26	0.3032 (5)	0.7066 (4)	0.6236 (3)	0.0845 (11)	
H26	0.3579	0.6821	0.6885	0.101*	
C27	0.3192 (5)	0.8298 (4)	0.6054 (3)	0.1023 (14)	
H27	0.3853	0.8896	0.6585	0.123*	
C15	0.0978 (6)	1.3295 (4)	0.2499 (4)	0.1108 (15)	
H3	0.0502	1.3314	0.3177	0.166*	
H2	0.0570	1.3872	0.2013	0.166*	
H1	0.2067	1.3539	0.2716	0.166*	
N1	0.1605 (2)	0.8740 (2)	0.25219 (18)	0.0489 (6)	
O1	0.0848 (2)	0.69988 (18)	0.10138 (16)	0.0578 (6)	

### Atomic displacement parameters (Å^2^)

**Table:**

	*U*^11^	*U*^22^	*U*^33^	*U*^12^	*U*^13^	*U*^23^
C1	0.0460 (15)	0.0516 (17)	0.0593 (16)	0.0120 (12)	0.0086 (13)	0.0048 (13)
C10	0.0449 (14)	0.0492 (16)	0.0540 (15)	0.0082 (11)	0.0119 (12)	0.0108 (12)
C16	0.0508 (15)	0.0450 (15)	0.0544 (15)	0.0123 (11)	0.0143 (13)	0.0080 (12)
C9	0.0489 (16)	0.0567 (18)	0.0652 (18)	0.0162 (13)	0.0125 (14)	0.0187 (14)
C11	0.0547 (16)	0.0477 (16)	0.0566 (16)	0.0118 (12)	0.0161 (13)	0.0024 (12)
C2	0.0490 (17)	0.0607 (19)	0.0671 (18)	0.0078 (13)	0.0032 (14)	−0.0033 (15)
C22	0.0513 (16)	0.0473 (16)	0.0521 (15)	0.0100 (12)	0.0067 (13)	0.0010 (12)
C4	0.0422 (15)	0.0596 (19)	0.082 (2)	0.0098 (13)	0.0107 (15)	0.0226 (16)
C23	0.0565 (16)	0.0527 (17)	0.0462 (14)	0.0079 (12)	0.0123 (13)	0.0020 (12)
C12	0.0645 (17)	0.0514 (17)	0.0589 (17)	0.0157 (13)	0.0144 (14)	0.0083 (13)
C21	0.0573 (18)	0.066 (2)	0.0652 (18)	0.0055 (14)	0.0170 (15)	0.0125 (15)
C3	0.0478 (17)	0.060 (2)	0.093 (2)	0.0083 (14)	−0.0060 (17)	0.0046 (17)
C8	0.0602 (18)	0.075 (2)	0.075 (2)	0.0158 (15)	0.0238 (16)	0.0203 (17)
C24	0.0505 (16)	0.0596 (19)	0.0590 (17)	0.0018 (13)	0.0138 (13)	0.0127 (14)
C17	0.0610 (18)	0.070 (2)	0.0643 (18)	0.0091 (15)	0.0206 (15)	0.0082 (15)
C5	0.0448 (17)	0.081 (2)	0.112 (3)	0.0058 (15)	0.0152 (19)	0.033 (2)
C13	0.090 (2)	0.0544 (19)	0.073 (2)	0.0154 (16)	0.0162 (18)	0.0122 (15)
C19	0.063 (2)	0.071 (2)	0.098 (3)	0.0118 (16)	0.041 (2)	0.0249 (19)
C18	0.094 (3)	0.088 (3)	0.067 (2)	0.018 (2)	0.038 (2)	0.0108 (18)
C7	0.068 (2)	0.107 (3)	0.094 (3)	0.025 (2)	0.044 (2)	0.036 (2)
C20	0.0578 (19)	0.069 (2)	0.090 (2)	0.0049 (15)	0.0180 (18)	0.0160 (18)
C14	0.125 (3)	0.066 (2)	0.097 (3)	0.034 (2)	0.033 (3)	0.024 (2)
C25	0.070 (2)	0.082 (2)	0.069 (2)	0.0103 (17)	0.0176 (18)	0.0276 (18)
C6	0.054 (2)	0.112 (3)	0.118 (3)	0.020 (2)	0.029 (2)	0.048 (3)
C28	0.111 (3)	0.062 (2)	0.068 (2)	0.0097 (19)	−0.001 (2)	−0.0060 (17)
C26	0.101 (3)	0.098 (3)	0.057 (2)	0.021 (2)	0.008 (2)	0.021 (2)
C27	0.130 (4)	0.096 (3)	0.064 (2)	0.018 (3)	−0.024 (2)	−0.015 (2)
C15	0.165 (4)	0.064 (3)	0.117 (3)	0.037 (3)	0.050 (3)	0.012 (2)
N1	0.0543 (13)	0.0453 (13)	0.0494 (12)	0.0119 (10)	0.0120 (10)	0.0063 (10)
O1	0.0514 (11)	0.0605 (13)	0.0576 (12)	0.0002 (9)	0.0120 (9)	−0.0051 (9)

### Geometric parameters (Å, °)

**Table:**

C1—O1	1.373 (3)	C24—C25	1.379 (4)
C1—C10	1.383 (4)	C24—H14	0.9300
C1—C2	1.414 (4)	C17—C18	1.399 (4)
C10—C9	1.432 (4)	C17—H15	0.9300
C10—C11	1.526 (4)	C5—C6	1.355 (5)
C16—C17	1.379 (4)	C5—H16	0.9300
C16—C21	1.386 (4)	C13—C14	1.485 (5)
C16—C22	1.506 (4)	C13—H17A	0.9700
C9—C8	1.411 (4)	C13—H17B	0.9700
C9—C4	1.425 (4)	C19—C20	1.364 (5)
C11—N1	1.477 (3)	C19—C18	1.368 (5)
C11—C23	1.520 (4)	C19—H18	0.9300
C11—H5	0.9800	C18—H19	0.9300
C2—C3	1.356 (4)	C7—C6	1.392 (5)
C2—H6	0.9300	C7—H20	0.9300
C22—N1	1.443 (3)	C20—H21	0.9300
C22—O1	1.456 (3)	C14—C15	1.508 (5)
C22—H7	0.9800	C14—H4	0.9700
C4—C3	1.406 (5)	C14—H22B	0.9700
C4—C5	1.424 (4)	C25—C26	1.356 (5)
C23—C24	1.369 (4)	C25—H23	0.9300
C23—C28	1.402 (4)	C6—H24	0.9300
C12—N1	1.477 (3)	C28—C27	1.396 (5)
C12—C13	1.519 (4)	C28—H25	0.9300
C12—H10A	0.9700	C26—C27	1.358 (6)
C12—H10B	0.9700	C26—H26	0.9300
C21—C20	1.379 (4)	C27—H27	0.9300
C21—H11	0.9300	C15—H3	0.9600
C3—H12	0.9300	C15—H2	0.9600
C8—C7	1.378 (4)	C15—H1	0.9600
C8—H13	0.9300		
			
O1—C1—C10	123.4 (2)	C18—C17—H15	119.8
O1—C1—C2	115.1 (2)	C6—C5—C4	120.4 (4)
C10—C1—C2	121.5 (3)	C6—C5—H16	119.8
C1—C10—C9	118.7 (3)	C4—C5—H16	119.8
C1—C10—C11	119.1 (2)	C14—C13—C12	114.1 (3)
C9—C10—C11	122.1 (2)	C14—C13—H17A	108.7
C17—C16—C21	117.9 (3)	C12—C13—H17A	108.7
C17—C16—C22	123.8 (3)	C14—C13—H17B	108.7
C21—C16—C22	118.3 (2)	C12—C13—H17B	108.7
C8—C9—C4	118.2 (3)	H17A—C13—H17B	107.6
C8—C9—C10	122.3 (3)	C20—C19—C18	119.7 (3)
C4—C9—C10	119.4 (3)	C20—C19—H18	120.1
N1—C11—C23	110.4 (2)	C18—C19—H18	120.1
N1—C11—C10	110.7 (2)	C19—C18—C17	120.4 (3)
C23—C11—C10	114.3 (2)	C19—C18—H19	119.8
N1—C11—H5	107.0	C17—C18—H19	119.8
C23—C11—H5	107.0	C8—C7—C6	120.3 (4)
C10—C11—H5	107.0	C8—C7—H20	119.8
C3—C2—C1	119.6 (3)	C6—C7—H20	119.8
C3—C2—H6	120.2	C19—C20—C21	120.1 (3)
C1—C2—H6	120.2	C19—C20—H21	120.0
N1—C22—O1	111.8 (2)	C21—C20—H21	120.0
N1—C22—C16	114.3 (2)	C13—C14—C15	114.6 (4)
O1—C22—C16	109.3 (2)	C13—C14—H4	108.6
N1—C22—H7	107.0	C15—C14—H4	108.6
O1—C22—H7	107.0	C13—C14—H22B	108.6
C16—C22—H7	107.0	C15—C14—H22B	108.6
C3—C4—C5	122.0 (3)	H4—C14—H22B	107.6
C3—C4—C9	118.9 (3)	C26—C25—C24	120.9 (3)
C5—C4—C9	119.2 (3)	C26—C25—H23	119.5
C24—C23—C28	117.5 (3)	C24—C25—H23	119.5
C24—C23—C11	123.8 (2)	C5—C6—C7	121.0 (3)
C28—C23—C11	118.6 (3)	C5—C6—H24	119.5
N1—C12—C13	112.2 (2)	C7—C6—H24	119.5
N1—C12—H10A	109.2	C27—C28—C23	119.6 (3)
C13—C12—H10A	109.2	C27—C28—H25	120.2
N1—C12—H10B	109.2	C23—C28—H25	120.2
C13—C12—H10B	109.2	C25—C26—C27	119.1 (3)
H10A—C12—H10B	107.9	C25—C26—H26	120.4
C20—C21—C16	121.6 (3)	C27—C26—H26	120.4
C20—C21—H11	119.2	C26—C27—C28	121.2 (4)
C16—C21—H11	119.2	C26—C27—H27	119.4
C2—C3—C4	121.8 (3)	C28—C27—H27	119.4
C2—C3—H12	119.1	C14—C15—H3	109.5
C4—C3—H12	119.1	C14—C15—H2	109.5
C7—C8—C9	120.9 (3)	H3—C15—H2	109.5
C7—C8—H13	119.6	C14—C15—H1	109.5
C9—C8—H13	119.6	H3—C15—H1	109.5
C23—C24—C25	121.6 (3)	H2—C15—H1	109.5
C23—C24—H14	119.2	C22—N1—C11	109.0 (2)
C25—C24—H14	119.2	C22—N1—C12	113.8 (2)
C16—C17—C18	120.3 (3)	C11—N1—C12	113.8 (2)
C16—C17—H15	119.8	C1—O1—C22	113.8 (2)
			
O1—C1—C10—C9	−179.0 (2)	C11—C23—C24—C25	178.0 (3)
C2—C1—C10—C9	0.9 (4)	C21—C16—C17—C18	0.5 (5)
O1—C1—C10—C11	3.4 (4)	C22—C16—C17—C18	−176.0 (3)
C2—C1—C10—C11	−176.7 (2)	C3—C4—C5—C6	179.2 (3)
C1—C10—C9—C8	178.9 (3)	C9—C4—C5—C6	−0.8 (5)
C11—C10—C9—C8	−3.6 (4)	N1—C12—C13—C14	−175.6 (3)
C1—C10—C9—C4	−0.3 (4)	C20—C19—C18—C17	−0.5 (5)
C11—C10—C9—C4	177.2 (2)	C16—C17—C18—C19	0.0 (5)
C1—C10—C11—N1	15.7 (3)	C9—C8—C7—C6	0.2 (5)
C9—C10—C11—N1	−161.8 (2)	C18—C19—C20—C21	0.3 (5)
C1—C10—C11—C23	−109.8 (3)	C16—C21—C20—C19	0.2 (5)
C9—C10—C11—C23	72.7 (3)	C12—C13—C14—C15	−177.4 (3)
O1—C1—C2—C3	179.4 (2)	C23—C24—C25—C26	−0.3 (5)
C10—C1—C2—C3	−0.5 (4)	C4—C5—C6—C7	1.2 (6)
C17—C16—C22—N1	−126.3 (3)	C8—C7—C6—C5	−0.9 (6)
C21—C16—C22—N1	57.3 (3)	C24—C23—C28—C27	−2.6 (5)
C17—C16—C22—O1	−0.2 (4)	C11—C23—C28—C27	−178.9 (3)
C21—C16—C22—O1	−176.6 (2)	C24—C25—C26—C27	−0.6 (6)
C8—C9—C4—C3	−179.9 (3)	C25—C26—C27—C28	−0.1 (7)
C10—C9—C4—C3	−0.6 (4)	C23—C28—C27—C26	1.8 (6)
C8—C9—C4—C5	0.1 (4)	O1—C22—N1—C11	66.5 (3)
C10—C9—C4—C5	179.4 (3)	C16—C22—N1—C11	−168.7 (2)
N1—C11—C23—C24	−114.6 (3)	O1—C22—N1—C12	−61.7 (3)
C10—C11—C23—C24	11.1 (4)	C16—C22—N1—C12	63.1 (3)
N1—C11—C23—C28	61.4 (3)	C23—C11—N1—C22	79.0 (3)
C10—C11—C23—C28	−172.9 (3)	C10—C11—N1—C22	−48.7 (3)
C17—C16—C21—C20	−0.6 (4)	C23—C11—N1—C12	−152.9 (2)
C22—C16—C21—C20	176.0 (3)	C10—C11—N1—C12	79.5 (3)
C1—C2—C3—C4	−0.5 (5)	C13—C12—N1—C22	−150.4 (3)
C5—C4—C3—C2	−179.0 (3)	C13—C12—N1—C11	84.0 (3)
C9—C4—C3—C2	1.1 (5)	C10—C1—O1—C22	11.7 (4)
C4—C9—C8—C7	0.2 (4)	C2—C1—O1—C22	−168.2 (2)
C10—C9—C8—C7	−179.1 (3)	N1—C22—O1—C1	−47.0 (3)
C28—C23—C24—C25	1.9 (4)	C16—C22—O1—C1	−174.6 (2)

### Hydrogen-bond geometry (Å, °)

**Table:**

*D*—H···*A*	*D*—H	H···*A*	*D*···*A*	*D*—H···*A*
C17—H15···O1	0.93	2.42	2.762 (4)	102

## References

[bb1] Alfonsov, V. A., Metlushka, K. E., McKenna, C. E., Kashemirov, B. A., Kataeva, O. N., Zheltukhin, V. F., Sadkova, D. N. & Dobrynin, A. B. (2007). *Synlett*, **3**, 488–490.

[bb2] Barker, M., Clackers, M., Copley, R., Demaine, D. A., Humphreys, D., Inglis, G. G. A., Johnston, M. J., Jones, H. T., Haase, M. V., House, D., Loiseau, R., Nisbet, L., Pacquet, F., Skone, P. A. & Shanahan, S. E. (2006). *J. Med. Chem.* **49**, 4216–4231. 10.1021/jm060302x 16821781

[bb3] Ji, M., Chen, H. & Miao, S. (2005). *Anal. Sci. X-ray Struct. Anal. Online*, **21**, x29.

[bb4] Peglion, J. L., Vian, J., Gourment, B., Despaux, N., Audinot, V. & Millan, M. (1997). *Bioorg. Med. Chem. Lett.* **7**, 881–886.

[bb5] Petterson, I., Liljefors, T. & Bodeso, K. (1990). *J. Med. Chem.* **33**, 2197–2204. 10.1021/jm00170a025 1973733

[bb6] Ren, H. Y., Grady, S., Gamenara, D., Heinzen, H., Moyna, P., Croft, S. L., Kendrick, H., Yardley, V. & Moyna, G. (2001). *Bioorg. Med. Chem. Lett.* **11**, 1851–1854. 10.1016/s0960-894x(01)00308-0 11459645

[bb7] Rigaku (2005). *CrystalClear* Rigaku Corporation, Tokyo, Japan.

[bb8] Sheldrick, G. M. (2008). *Acta Cryst.* A**64**, 112–122. 10.1107/S0108767307043930 18156677

